# Kumar and Daigavane's Impacted Maxillary Canine Grading System: A New Classification System For Treatment of Cleft Lip and Palate

**DOI:** 10.7759/cureus.29586

**Published:** 2022-09-26

**Authors:** Nikhil Kumar, Pallavi Daigavane

**Affiliations:** 1 Orthodontics and Dentofacial Orthopaedics, Sharad Pawar Dental College, Wardha, IND

**Keywords:** cleft lip & palate, unilateral cleft lip and palate, canine classification, grading system, impacted canine

## Abstract

Introduction

Maxillary canines are the most commonly impacted teeth, second only to third molars. Cleft lip and palate patients have a higher chance of impacted canines due to defects in bone formation and the soft tissue enclosing it. Various authors have used two-dimensional radiographs and developed grading systems to streamline treatment modalities to deal with the impacted canines.

Material and Methods

A study was conducted in the Department of Orthodontics and Dentofacial Orthopaedics. Children aged nine to 12 were selected for this evaluation based on both clinical and radiographic examination. Four different parameters were set to evaluate the impacted canine location. which was based on angulation, vertical height, root apex location, and defect area involved.

Result

Based on these evaluations, a classification was developed by the present authors, and later the cases were assessed as per the new system that we named Kumar and Daigavane's (KD) Impacted Maxillary Canine Grading System which we developed to give us more clarity for treatment and prognosis for treatment of cleft lip and palate.

Conclusion

A three-dimensional evaluation is also advised for a better understanding of the impacted canines. Many of the shortcomings of the two-dimensional assessment can be diminished. This new grading system will improve cleft diagnosis and prognosis in patients with cleft lips and/or palates.

## Introduction

The development of a tooth is slowed or stopped as a result of impaction. Various terms, including delayed eruption, primary retention, submerged teeth, impacted teeth, and so on, are used to describe impaction in the scientific literature. A canine is considered impacted if its eruption is halted after the root has formed completely, or if the contralateral tooth has erupted for at least six months after the same stage of development [1]. The maxillary canines are the most difficult teeth to erupt because they develop laterally to the pyriform fossa and have the longest and most complex path to their final position in occlusion. Coulter and Richardson et al. [2] found that between the ages of five and 15, the maxillary canines move nearly 22 millimetres (mm) in three spatial dimensions. At the age of three, the maxillary canine is located high in the maxilla with its crown pointing mesially and lingually. At around age eight, it begins to angulate medially, with its crown located distally and slightly buccal to the lateral incisor [3]. From its original lingual position at the root apex of its deciduous ancestor, the canine now moves buccally. If it cannot move from the palatal to the buccal side, it will remain impacted on the palatal side [4]. The maxillary canines reposition themselves by first moving mesially until they reach the distal aspect of the lateral incisor root and then rising to a more vertical position by moving towards the occlusal plane. However, maxillary canines often erupt into the mouth at a pronounced mesial inclination [5].

In the case of congenitally absent lateral incisors, the canine may erupt mesially until it makes contact with the distal aspect of the central incisor root and emerges into the lateral incisor space. Therefore, upper permanent canines rely heavily on the guidance provided by the lateral incisor roots. Finding out which teeth are impacted is the most important part of treating clefts. This is due to the fact that canines are the most susceptible to damage, and their condition can be exacerbated by a wide variety of causes. The following are some of the clinical manifestations of canine impaction: incomplete eruption of the permanent canine or prolonged retention of the deciduous canine; inability to locate canine position through intraoral palpation of the alveolar process; absence of a normal labial canine bulge; presence of a palatal bulge; and delayed eruption, distal tipping, or migration of the canine. Maxillary canines that become impacted can be assisted in erupting and moving to the appropriate position in the dental arch with the help of surgery and orthodontics if they are detected early and treated promptly. In order to facilitate the treatment of affected canines, many authors have devised grading systems based on this two-dimensional radiograph. Orthopantomagram (OPG) had been used as a diagnostic tool for locating the impacted canine location by various authors.

A number of authors in this field have been cited in the literature. The affected canine position can be reliably predicted based on the sector classification. Using OPG, the sector drew several reference lines in order to identify the faulty canine component. The absence of a craniofacial anomaly supports the use of this classification scheme. consistent with cleft cases. The first to include angular and linear measurements for the impacted canine in addition to vertical and horizontal ones were Ericson and Kurol et al. [6-11] canine cusp tip distance to occlusal plane (d-distance), canine impact angle (α-angle), and canine sagittal split (s-sector) (sector 1, between the midline and the axis of the central incisor; sector 2, between the axis of the central and lateral incisors; or sector 3, between the axis of the lateral and central incisors) [12-14]. These factors determine the likelihood of an impacted tooth, the time required for orthodontic traction, and the intensity of treatment necessary to move an impacted tooth. In most cases, the panoramic radiograph variables will dictate where the canine tooth will be located in a cleft patient's mouth. This technique helped pinpoint the spot where the canine was impacted, but it didn't deal with the big picture. Because of the patient's midfacial discrepancy and lack of a central incisor, it was impossible to record the treatment modality. Without a straight edge to use as a starting point, calculating angles was also extremely difficult [15,16]. In order to improve treatment planning, it was necessary to evaluate the range of canine positions in the cleft and to institute a new grading system for CLP cases. 

This grading system, developed by the authors Kumar and Daigavane, is specifically framed for cleft lip and palate cases where there is a scarcity of the born breach in the continuous alveolar ridge, absence or congenitally missing of anterior teeth that leads to the impaction of canine. The reason for the new grading system is that sector classification for impacted canines is used worldwide for non-cleft patients. The parameter for grading impacted canine is the midline of the central incisor and lateral incisor, the long axis of the tooth. In unilateral cleft lip and palate or bilateral cleft lip and palate cases this is not applicable due to a defect of the cleft area and missing or congenitally missing lateral incisor, so sector classification will provide the wrong prognosis for the impacted canine. The new grading system used bone defect area in grading as well as angulation of the canine and the vertical height in maxillary bone is measured from the occlusion plane. The degree and severity of malocclusion are more severe in cleft than in non-cleft patients. So there was an urgent need for a new grading system for impacted maxillary canine in patients with cleft lip and palate cases.

## Materials and methods

A study was conducted in the Department of Orthodontic and Dentofacial Orthopaedics, Sharad Pawar Dental College, Sawangi, Wardha, in collaboration with the Department of Oral Medicine and Radiology. The ethical committee of the Datta Meghe Institute of Medical Sciences approved the study (Ref no. DMIMS (DU)/IEC/2020-21/260). The CLP patients were selected from the Smile Train outpatient department. The localization of the maxillary canine was based on both clinical as well as radiographic examination:

For clinical examination, the following features were taken into consideration during evaluation: (1) absence of a normal labial canine bulge; in other words, either inability to locate canine position through intraoral palpation of the alveolar process or the presence of an asymmetry in the canine bulge noted during alveolar palpation; and (2) the presence of a palatal bulge. For the radiographic examination, a panoramic view was taken for evaluation of canine angulation, height, root apex position, and exposure of canine in the cleft defect.

Canine Angulation

**Figure 1:**
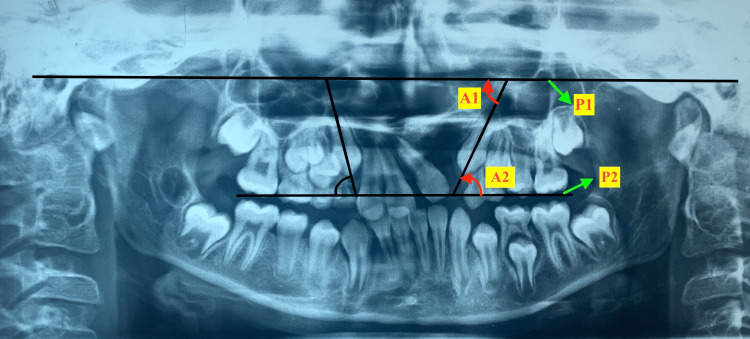
Canine angulation

The FH (Frankfurt horizontal) plane was constructed on OPG by drawing a line from orbital to porion on the right side and continuing to the left side, considered as a P1 reference plane, and the inner angle was measured between the long axis of the canine to the FH plane, considered as an A1 angle. The occlusion plane was constructed on OPG by drawing a line from the cusp tip of a maxillary molar on the right side to the maxillary molar on the left side, considered as a P2 reference plane, and the inner angle was measured between the long axis of the canine to the Occlusion plane, considered as an A2 angle (Figure 1).

Vertical height

**Figure 2:**
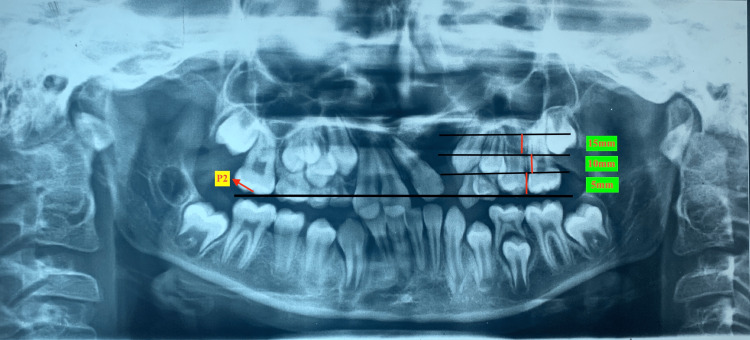
Vertical height

The vertical height was measured from the occlusion plane to the canine cusp tip. From the occlusion plane, a vertical height of 5 mm shows that the canine cusp tip is at the cervical edge of the crown. A vertical height of 10 mm shows that the canine cusp tip is in the middle third of the root of the tooth. A vertical height of 15 mm or more shows that the canine cusp tip is in the most apical position (Figure 2).

Canine apex root position in the horizontal plane

**Figure 3:**
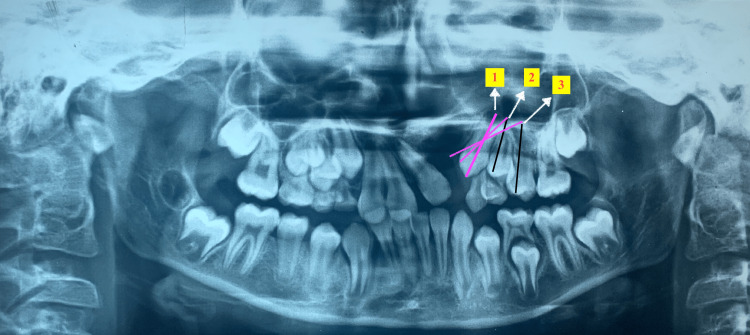
Canine root apex position The long axis of the canine is shown by a pink line coinciding with the long axis of the canine (point 1), 1st premolar (point 2), and 2nd premolar (point 3).

In this parameter, the position of the root apex of the canine was graded accordingly: (1) above the canine position; (2) above the 1st premolar; (3) above the 2nd premolar; (4) toward the lateral incisor. This coincided with the angulation of the canine with P1 and P2. The long axis of the canine is shown by a pink line coinciding with the long axis of the canine (point 1), 1st premolar (point 2), and 2nd premolar (point 3) (Figure 3).

Canine exposure to the cleft defect

**Figure 4:**
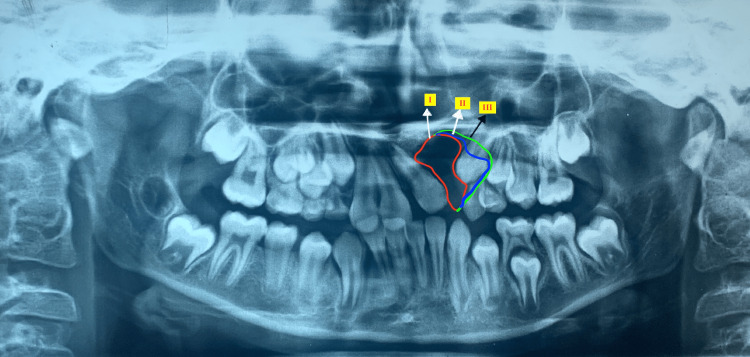
Canine exposure in the cleft defect Grade I is shown in red, Grade II in blue, and Grade III in green.

The outer border of the cleft defect area was drawn on OPG and the positions of canines within the cleft defect area were graded accordingly. Grade I is shown in red, Grade II in blue, and Grade III in green (Figure 4).

## Results

Based on these evaluations, A classification was set and later the cases were assessed as per the new classification system that gave us more clear onset (Table 1).

**Table 1:** Kumar and Daigavane’s impacted canine chart for cleft lip and palate

	Grade I	Grade II	Grade III	Grade IV
Clinical finding	Presence of labial or palatal canine bulge at the alveolar process	Presence of Labial canine bulge above alveolar process and till the depth of vestibule and in palatal region palatal bulge is seen in between the rugae area	Presence of labial bulge above the depth of vestibule and palatal bulge beyond rugae area	Absence of any labial or palatal canine bulge
Angulation measured from the Frankfurt horizontal plane (A1) and occlusion plane (A2)	90^0^ ± 10^0^	45^0^ ± 10^0^	180^0^ ± 10^0^ and above	Distally angulated
Vertical height measured from occlusion plane	5 mm ± 2 mm	10 mm ± 2 mm	15 mm ± 2 mm	Above 15 mm
Apex location	Above canine position	Above 1st premolar	Above 2nd premolar	Towards lateral incisor
Exposure to cleft defect	Bone lining at the tooth	Only crown in the defect area	Crown and half root in the defect area	Only one-fourth apex in the bone
Inference	Good prognosis for canine eruption and alignment	Average prognosis for canine eruption and alignment	Poor prognosis for canine eruption alignment	The hopeless prognosis for canine eruption alignment

## Discussion

Grading the impacted canine as Grade I

On clinical examination, there will be the presence of a labial or palatal canine bulge at the alveolar process. The radiographic evaluation depicts a 90°±10° angulation of A1 and A2 measured from P1 and P2, suggesting that the canines are erect and stable on the occlusion plane. The vertical height of the impacted canine crown will be at a distance of 5 mm ±2 mm when measured from P2. The location of the root apex of the canine will be along the long axis of the canine. The tooth meets in the cleft defect but is completely impacted into the bone, leaving no exposure in the cleft defect. On observing these findings, it can be stated that the prognosis of this grade will be good for eruption and alignment of canines. As part of the suggested treatment plan, a soft-tissue window will be made and a thin layer of bone covering the crown of the canine will be removed. This will help the canine fit better in the occlusion.

Grading the impacted canine as Grade II

On clinical examination, there will be the presence of a labial canine bulge above the alveolar process and till the depth of the vestibule and in the palatal region, a palatal bulge is seen in the area between the rugae. The radiographic evaluation depicts a 45°±10° angulation of A1 and A2 measured from P1 and P2. Now, this can be near the lateral and away from the lateral incisor. The vertical height of the impacted canine crown will be at a distance of 10 mm ± 2 mm when measured from P2. The location of the root apex of the canine will be near the first premolar. Concerning the cleft defect area, the only crown of the impacted canine will be seen in the bony defect area when fully covered with the soft tissue of the palate. On observing these findings, it can be stated that the prognosis of this grade will be average for eruption and alignment of canine. The suggested treatment plan will be age-specific. Canine eruptions should be monitored with ABG before the age of 10-12 years. After 12 years of age, surgical exposure of the impacted canine and orthodontics adjusts with the alignment of the root in the bone near the cleft to utilise the graft.

Grading the impacted canine as Grade III

On clinical examination, there will be the presence of a labial bulge above the depth of the vestibule and a palatal bulge beyond the rugae area. The radiographic evaluation depicts a 180°± 10°and above angulation of A1 and A2 measured from P1 and P2. The vertical height of the impacted canine crown will be at a distance of 15 mm ± 2 mm when measured from P2. The location of the apex will be above the 2nd premolar. Concerning the cleft defect area, the crown and 12 roots will be seen in the bony defect when fully covered with the soft tissue of the palate. On observing these findings, it can be stated that the prognosis of this grade will be poor for eruption and alignment of canines. The suggested treatment plan will be age-specific. ABG should be done with monitoring canine eruptions before 10-12 years. After 12 years of age, surgical exposure of impacted canine and orthodontics adjusts with the alignment of the root in the bone near the cleft to utilise the graft.

Grading the impacted canine as Grade IV

On clinical examination, there will be an absence of any labial or palatal canine bulge. The radiographic evaluation depicts the distal angulation of A1 and A2 measured from P1 and P2. The vertical height of the impacted canine crown will be at a distance of above 15mm when measured from P2. The location of the apex will be towards the lateral incisors. Concerning the cleft defect area, only the 14th root apex of the impacted canine is in the bone while the rest will be lying in the bony defect area and covered with the soft tissue of the palate. On observing these findings, it can be stated that the prognosis of this grade will be hopeless for eruption and alignment of canines. In order to save time and get the functional occlusion done faster, the most likely treatment plan will be surgical extraction instead of trying to align the teeth.

## Conclusions

We can gain insight into the prognosis and plan the treatment protocol for these cases by implementing a more recent grading system for the evaluation of the impacted canine in CLP cases. We anticipate a swift approach to treating adult cleft cases with this new grading system. A three-dimensional evaluation is also advised for a better understanding of the impacted canines and their relationship to the surrounding structure. Many of the shortcomings of the two-dimensional assessment can be diminished by the three-dimensional evaluation of CBCT. However, since three-dimensional diagnosis units might not be available at all of the operational centers, this two-dimensional OPG-based evaluation will assist medical professionals in developing a plan for how to treat patients with cleft lips and/or palates. 
